# Biological validation of faecal corticosterone metabolites as a non-invasive stress assessment in translocated California valley quail (*Callipepla californica*)

**DOI:** 10.1093/conphys/coae012

**Published:** 2024-04-13

**Authors:** Sarah A Currier, Jeffrey G Whitt, Kelly S Reyna

**Affiliations:** The Quail Research Laboratory, Ted and Donna Lyon Center for Gamebird Research, Texas A&M University-Commerce, Commerce, TX, 75429, USA; The Quail Research Laboratory, Ted and Donna Lyon Center for Gamebird Research, Texas A&M University-Commerce, Commerce, TX, 75429, USA; The Quail Research Laboratory, Ted and Donna Lyon Center for Gamebird Research, Texas A&M University-Commerce, Commerce, TX, 75429, USA

**Keywords:** biological validation, California valley quail, conservation, corticosterone, faecal corticosterone metabolites, physiology, population restoration, stress hormones, translocation

## Abstract

US quail species are vulnerable to population declines as a result of climate change, habitat loss and habitat fragmentation, all of which can result in physiological stress. Additionally, population restoration techniques (PRTs), like translocations, also induce stress. Traditional assessments of avian stress hormone levels include capturing and handling birds to extract blood, methods that are inherently stressful and can compound stress analyses. However, the stress hormone corticosterone (CORT) is metabolized from the blood and excreted in faeces as faecal corticosterone metabolites (FCMs). FCMs have been used as a non-invasive measurement of stress hormone levels in a variety of species, but must be validated for each species. The objective of this study was to biologically validate the use of FCMs as a non-invasive measurement of CORT levels in California valley quail (*Callipepla californica*). Reference and treatment quail were acclimated for 3 weeks in an outdoor aviary. Subsequently, treatment quail were subjected to a simulated 48-h translocation, a common and stress hormone-inducing PRT. Faecal samples were collected every 4 h and processed using an enzyme immunoassay. Mean FCM concentrations of treatment quail (41.50 ± 16.13 ng/g) were higher than reference FCM concentrations (24.07 ± 10.4 ng/g). These results biologically validate the use of FCMs as a non-invasive method to assess CORT levels in California valley quail, demonstrate diurnal variation in quail CORT levels, and confirm that quail translocations are a stress-inducing PRT. Ultimately, this research validates a new non-invasive tool for stress response measurement to advance quail research, management and conservation.

## Teaser

This study biologically validates the use of faecal corticosterone metabolites as a non-invasive method for detecting stress in quail, demonstrates diurnal variation in quail stress levels, confirms that translocations elevate stress, which likely impacts success, and establishes a new non-invasive tool for stress measurement in quail research, management and conservation.

## Introduction

Stress assessments are often overlooked in US quail population declines and population restoration. US quail populations are vulnerable to decline as a result of unpredictable stressors like extreme weather, climate change (Guthery *et al.,* 2000; Reyna and Burggren, 2017; Tanner *et al.,* 2017; Reyna, 2019; Wilsey *et al.,* 2019; Whitt and Reyna, 2022), habitat loss and habitat fragmentation (Brennan and Covington, 1994; Hernández *et al.,* 2013; Pope and Heekin, 2017), all of which induce physiological stress responses. For example, elevated stress hormone levels have been observed in birds as a result of anthropogenic disturbances (Thiel *et al.,* 2011; Jankowski *et al.,* 2014; Arlettaz *et al.,* 2015) and extreme climatic events (Wingfield *et al.,* 2017), which are expected to increase in frequency and severity due to global climate change (Seneviratne *et al.,* 2012; Masson-Delmotte *et al.,* 2021). Climate change can also exacerbate the effects of other stressors (Felton *et al.,* 2009; Şekercioğlu *et al.,* 2012).

Quail respond to stressors using two pathways: (1) a rapid, short-term response, mediated by the autonomic nervous system, which involves a rapid release of catecholamine from the adrenal medulla, and (2) a slower, long-term response, mediated by the hypothalamic–pituitary–adrenal (HPA) axis, which involves the release of corticosterone (CORT, 17-deoxycortisol) from the adrenal cortex (Creel, 2001; Suorsa *et al.,* 2003; Tempel and Gutiérrez, 2004; Cockrem, 2007, 2013; Sheriff *et al.,* 2011; Wingfield *et al.,* 2017; Palme, 2019; Romero and Gormally, 2019; van Vliet *et al.,* 2020). As a stress response, elevated CORT levels act as a catalyst that transitions quail into survival mode whilst suppressing nonessential functions. However, long-term elevated CORT levels disrupt normal bodily functions and can lead to disease and death (Blas and Fairhurst, 2022). Accordingly, CORT’s role in the stress response, and the detectability of its metabolites using non-invasive methods, make it a common proxy for assessing the magnitude of an animal’s stress response (Sheriff *et al.,* 2010; Ellis *et al.,* 2012).

Prolonged or repeated exposure to stressors can directly influence bird populations by altering foraging, predator avoidance, fledging and reproduction, (Cyr and Romero, 2007; Crespi *et al.,* 2013; Martin *et al.,* 2017; Wingfield *et al.,* 2017). Stressor persistence can induce a type 1 allostatic overload, where energy demand exceeds energy availability (Blas and Fairhurst, 2022), and birds decrease or eliminate behaviours (e.g. reproduction) not immediately necessary for survival (McEwen and Wingfield, 2003). Reproduction may be interrupted through several different, but complimentary pathways, including increasing production of gonadotropin-inhibitory hormone, reducing production of gonadotropin-releasing hormone (GnRH), and decreasing sensitivity of pituitary gonadotrope cells to GnRH and of ovaries to luteinizing hormone (Sapolsky *et al.,* 2000; Wingfield and Sapolsky, 2003; Dickens and Bentley, 2014; Son *et al.,* 2014). For example, capturing and holding wild European starlings (*Sturnus vulgaris*) blocked a time-sensitive seasonal increase in HPA axis activity, preventing the transition from early gonadal development to an active breeding state, which inhibited breeding (Dickens and Bentley, 2014). The resultant reduction in reproduction in birds contributes to population declines.

Chronic stress can also disrupt acute stress responses, including the fight or flight response, due to a decrease in the sensitivity of the HPA axis (Rich and Romero, 2005; Romero and Butler, 2007; Dickens *et al.,* 2009). For example, European starlings caught in the wild and placed into captivity lost the ability to produce a fight or flight response when exposed to a loud noise as a stressor (Dickens and Romero, 2009). In addition, when the fight or flight response was chronically stimulated, it negatively impacted cardiovascular health, leading to hypertension due to surplus catecholamine exposure (Rupp, 1999). Population restoration techniques (PRTs), like translocations, are also stressors that can increase CORT levels. US quail species are economically important gamebirds (Johnson *et al.,* 2012; Wszola *et al.,* 2020) and part of a $3.7 billion per year upland gamebird hunting industry (Sportsmen’s Alliance, 2021). For >150 years, translocations have been used in attempts to bolster populations (Hernández *et al.,* 2013; Gomez and Reyna, 2017; Whitt *et al.,* 2017). Quail translocations are rarely successful (Perez *et al.,* 2002; Scott *et al.,* 2013; Whitt *et al.,* 2017), especially when used for reintroduction (Martin *et al.,* 2017). Despite translocations rarely having long-term success, there is increasing interest in translocation of wild quail (Stephenson *et al.,* 2011; Martin *et al.,* 2017; Sisson *et al.*, 2017). Numerous hypotheses have been proposed for the low success rate (Sokos *et al.,* 2008; Martin *et al.,* 2017), but little is known about how stress influences translocation success.

Translocation consists of capturing, handling, holding, transporting and releasing quail to a novel site, each of which can result in acute and chronic stress, independent of other processes (Buchanan, 2000; Fazio and Ferlazzo, 2003; Jones *et al.,* 2005; Teixeira *et al.,* 2007; Dickens *et al.,* 2009, 2010; Wingfield and Romero, 2011; Batson *et al.,* 2017; Martin *et al.,* 2017). For example, short translocations may induce an acute stress response; however, the duration of a quail translocation is typically 1–3 days, which increases the occurrence of a chronic stress response as indicated by chronically elevated CORT levels (Dickens *et al.,* 2010). Both chronic and acute stress responses can inhibit survival of translocated animals through increased energy requirements, capture myopathy, dispersal distance and weight loss (Armstrong and Seddon, 2008; Dickens *et al.,* 2010; Breed *et al.,* 2019), which lead to a failure of the translocation by increasing susceptibility to disease and death (Dickens *et al.,* 2010; Martin *et al.,* 2017).

Weight loss has been observed when translocating wild birds to novel environments (Rich and Romero, 2005; Dickens *et al.,* 2009; Fischer *et al.,* 2018), and is a response to chronic stress. For example, European starlings lost 5–15% of their body weight after being exposed to a 14-day regime of multiple stressors during a translocation (Awerman and Romero, 2010). California valley quail (*C. californica*) exhibited a mean weight loss of 14.3% when translocated from Idaho to Texas (Reyna *et al.,* 2020). This is important because body mass can influence survival and the overall success of translocations. For example, Cirl buntings (*Emberiza cirlus*) with a higher body weight at capture were more likely to survive in their new habitat than those with lower body weights (Fountain *et al.,* 2017). Reducing stressors during translocation could reduce weight loss and increase post-release survival (Warwick *et al.,* 2006).

The traditional method of measuring stress in birds requires capturing, handling and extracting blood samples, inherently stress-inducing actions (Arnold *et al.,* 2008), followed by measuring blood CORT levels. It is assumed that if this process occurs within 3 min of initial handling, CORT levels will be indicative of the physiological condition of the bird prior and will not represent the elevated stress incurred during the procedure (Littin and Cockrem, 2001; Cockrem and Silverin, 2002). However, CORT levels quickly rise during handling and peak within 15–30 min. In addition, handling associated with blood extractions adds to the cumulative stress experienced by the bird, and nearby birds, independent of the extraction (Dickens *et al.,* 2010; O’Dell *et al.,* 2014; Wein *et al.,* 2017).

Steroid hormones, like CORT, can also be measured from urine, faeces, hair and feather samples (Bortolotti *et al.,* 2008; Sheriff *et al.,* 2011). One effective, non-invasive and increasingly popular method to evaluate a stress response is by measuring faecal corticosterone metabolites (FCMs; Romero and Remage-Healey, 2000; Tempel and Gutiérrez, 2004; Möstl *et al.,* 2005; Dickens *et al.,* 2009; Sheriff *et al.,* 2010). Measuring FCMs as an assessment of CORT levels has been successful in a wide range of avian studies (Washburn *et al.,* 2003; Fletcher *et al.,* 2018; Sokół and Koziatek-Sadłowska, 2020). Excretion of FCMs varies between species, as does the suitability of different immunoassays to accurately detect CORT; therefore methods must be validated for each species (Palme *et al.,* 1996; Wasser *et al.,* 2000; Palme, 2019). Physiological validation of measuring FCM concentrations requires injecting a known quantity of CORT into an animal (Touma and Palme, 2005), an invasive and stressful procedure (Palme, 2019; Mohlman *et al.,* 2020). Biological validation of measuring FCM concentrations is a non-invasive procedure where faeces is collected before and after an event that increases HPA activity for an extended time, resulting in increased blood CORT levels (Touma and Palme, 2005; Palme, 2019).

The goal of this study was to biologically validate the use of FCMs as a non-invasive assessment of CORT levels in California valley quail using a simulated translocation as the stressor. This is the first biological validation of FCMs as a tool to detect elevated CORT levels in a new world quail species. Mohlman *et al.* (2020) performed a physiological validation of FCMs with northern bobwhite (*Colinus virginianus*), another new world quail, but were unsuccessful at a biological validation due to an ineffective biological stressor.

## Materials and Methods

Flight-ready captive-reared valley quail (*n* = 63 females, *n* = 57 males), 16–24 weeks in age, were acquired from a breeder (ZKD Game Birds, West Point, TX, USA) in October, 2020. Thirteen valley quail (*n* = 5 females, *n* = 7 males) were selected at random to use for biological validation, with the remainder used for diurnal FCM analysis. Quail were housed and acclimated in groups in an outdoor aviary (Quail Hotel, Fannin Fabrication, Bonham, TX, USA), where they were provided with natural perches and dust baths for 3 weeks (Hawkins *et al.,* 2001). Mean temperature during this period was 16.4°C. Birds were provided with gamebird feed (Gamebird Starter and Grower, M-G Inc., Weimar, TX, USA) and water *ad libitum*. This mimicked the time and conditions in which quail were held prior to the first shipment of trapped birds during a 2019 translocation of valley quail (Reyna *et al.,* 2020). Acclimation was assumed when birds exhibited typical behaviour (e.g. eating, drinking, roosting, grooming; Reyna and Newman, 2018).

To biologically validate FCMs in valley quail, reference (low stress) faecal samples were obtained from acclimated valley quail. The floor of the aviary was lined with a plastic sheet. The birds were observed from ~50 m away with binoculars to record the sex of the bird, and to ensure collected faecal samples were fresh (<1 h old) and uncontaminated. Once collected, individual faecal samples were placed into a plastic storage bag (Ziploc freezer quart, S. C. Johnson & Son, Inc., Racine, WI) and labelled with the date, time and sex of the bird. Because individual birds could not be matched to collected faecal samples, reference sample data were pooled following FCM analysis. Ambient temperature, relative humidity and presence of direct sunlight were recorded on the plastic storage bag, since these factors can degrade hormone detection probability (Shipley *et al.,* 2019). Immediately after collection and labelling, samples were stored in a freezer at −20°C until FCM analysis (Wasser *et al.,* 1988; Messmann *et al.,* 1999; Khan *et al.,* 2002).

For biological validation, quail were transported to the animal care facility at Texas A&M University-Commerce to undergo a simulated 48-h translocation, a stressful event (Martin *et al.,* 2017). Each quail was given a unique leg band and had their age, sex, and weight recorded. Quail were held individually in research-approved breeding pens located in a temperature- (20 ± 2°C), humidity- (45 ± 15%) and light-controlled room with a 12 L:12 D photoperiod. To ensure accuracy and reduce contamination, faecal samples were collected from the underlying waste pan trays 4–5 times daily (Millspaugh and Washburn, 2004; Mohlman *et al.,* 2020). The frequency of faeces collection ensured a representative sample, since FCMs may fluctuate diurnally (Breuner *et al.,* 1999). Individual faecal samples were placed in a plastic storage bag and labelled with the bird ID number, sex, time, and date of collection. Steroid hormone concentrations can be affected by storage duration (Millspaugh and Washburn, 2004) and bacteria (Lexen *et al.,* 2008). Therefore, treatment faecal samples were immediately stored in a freezer (−20°C) until FCM analysis (Wasser *et al.,* 1988; Messmann *et al.,* 1999).

FCM concentrations of reference and treatment quail faecal samples were measured using an Enzyme Immunoassay (EIA) Kit (K-014H, Arbor Assays, Ann Arbor, MI, USA). The kit was validated for use on CORT extracted from dry faecal extracts on a multi-species design (DeVries and Jawor, 2013; Fletcher *et al.,* 2018), and included detailed instructions for extracting CORT from faecal samples and analysing FCM concentrations. To determine the assays suitability for valley quail, 10 faecal extracts were pooled from the valley quail simulated translocation, and a serial dilution (*n* = 6 dilutions) was performed. FCM concentrations from the pooled faecal extracts were parallel to the slope of the standard curve, verifying suitability for valley quail. The sensitivity of the EIA was determined to be 18.19 pg/ml. Optical density was calculated using a Synergy LX Multi-mode microplate reader (Bio Tek Instruments, USA), and entered into MyAssay web (https://www.myassay.com), to calculate FCM concentration in picograms per millilitre. FCM concentrations reported in picograms per millilitre were converted to nanogramme/gramme for comparison to other studies.

All animals were handled in accordance with procedures outlined in the Guide for the Care and Use of Laboratory Animals (National Research Council, 2010), and Texas A&M University-Commerce Animal Use Protocol P20–013.

### Statistical analysis

Because birds could not reliably be distinguished without technicians approaching close enough to elicit visible agitation and warning calls from the reference birds, we pooled samples for statistical comparison. All statistical analyses were conducted in R (version 4.0.2, R Foundation, Austria). Shapiro–Wilk’s statistical test was used to check for normality (Zar, 1996). Welch’s 2-sample *t*-test was used to calculate the *P*-value, mean and 95% confidence interval between 2 variables with normal distributions. Mann–Whitney *U*-test was used to compare 2 variables with non-normal distributions. Kruskal–Wallis test was used to compare multiple variables with non-normal distributions, followed by Dunn’s test with Bonferroni correction for pairwise comparisons. Correlations were tested using Pearson’s product–moment correlation with Bonferroni correction (Zar, 1996). All weight data are presented as mean ± standard deviation (SD). Results were significant at alpha < 0.05.

## Results

**Figure 1:**
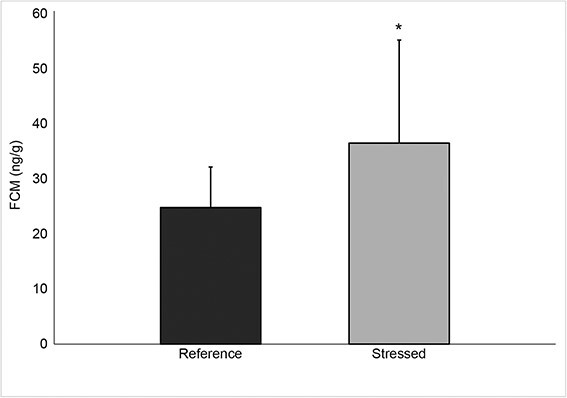
**Biological validation of using FCMs in translocated California valley quail.** FCM concentrations (mean ± SD) were extracted from faeces collected from acclimated quail (*n* = 13 birds, 27 samples) prior to (Reference) and during a 48-h simulated translocation (treatment, 70 samples). FCM concentrations from treatment birds were higher than reference FCM concentrations (Mann–Whitney *U*, *Z* = 4.20, *P* < 0.001). Asterisk indicates statistical difference.

Mean ± SD FCM concentrations (*n* = 13 quail; 70 faecal samples) of valley quail during the simulated translocation were 41.50 ± 16.13 ng/g, and higher than reference FCM concentrations (24.07 ± 10.4 ng/g; *n* = 13 quail; 27 faecal samples; Mann–Whitney *U*, *Z* = 4.20, *P* < 0.001; Fig. 1). Mean 48-h weight loss was 17.8 ± 6.3 g, or 12 ± 4.4% of initial body mass.

Valley quail with higher initial body mass prior to the simulated translocation experienced lower mean FCM concentrations (r^2^ = 0.14, *P* < 0.001), and lower percentage of initial mass lost (r^2^ = 0.13, *P* < 0.001) during the simulated translocation. FCM concentrations positively correlated with percent of initial body mass loss (r^2^ = 0.27, *P* < 0.001). No difference in FCM concentrations (Mann–Whitney *U*, *Z* = 0.15, *P =* 0.44), total mass lost (Welch’s *t*-test, *P* = 0.58) or percentage of initial mass lost (Welch’s *t*-test, *P* = 0.29) was observed based on sex.

**Figure 2:**
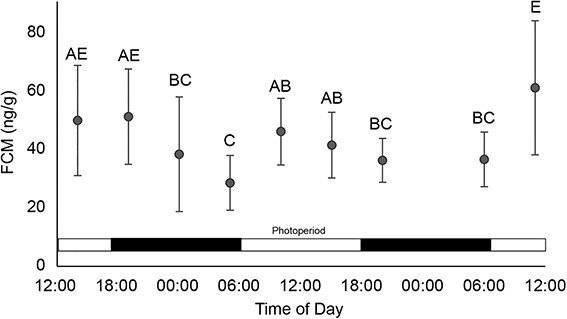
**Diurnal variation in FCM concentrations from California valley quail during a simulated 48-h translocation.** Treatment FCM concentrations correlated with photoperiod (12 L:12 D) and quail circadian rhythm. Letters indicate statistical groupings. Faecal sample numbers ranged from 9 to 37 per hour due to faecal dropping frequency.

Valley quail FCMs varied diurnally during the simulated translocation (Fig. 2), with higher concentrations from 10:00 to 18:00 and lower concentrations from 20:00 to 06:00 (Kruskal–Wallis, H = 62.3, *P* < 0.001). Mean FCM concentrations were not different between the first 24 h and last 24 h (Mann–Whitney *U*, *Z* = 1.86, *P* = 0.32).

## Discussion

This study was the first biological validation of FCMs as a non-invasive assessment of a stress response for a new world quail species. When exposed to a simulated translocation, valley quail experienced a 72.4% increase in FCMs compared to reference concentrations, indicating that this protocol successfully detected an increase in FCMs during a stressful event. These results are comparable to the 73% increase in FCMs recorded in northern bobwhites, another new world quail, during a physiological validation (Mohlman *et al.,* 2020). However, the increase in FCM levels recorded for both of these new world quail is lower than FCM levels recorded in other avian species. This is not surprising because excretions of metabolites can vary between species (Palme *et al.,* 1996; Wasser *et al.,* 2000; Palme, 2019). For example, experimentally stressed European starlings showed a ~100% increase in FCMs (Cyr and Romero, 2008). Following capture and holding for a veterinary examination, African penguins (*Spheniscus demersus*) showed a 155–349% increase in FCMs. Wild Dickcissels (*Spiza americana*) experienced a 1700% increase in FCM concentrations within 24 h of having leg harness transmitters attached (Suedkamp Wells *et al.,* 2003). Valley quail and northern bobwhite quail are both new world quail, and their similar stress response during validation, and variation from other taxa, was expected. This emphasizes the need to validate FCM for individual species.

The correlation of weight loss with FCM levels further supports the association of chronic stress with weight loss in birds (Cyr and Romero, 2007; Dickens *et al.,* 2009; Awerman and Romero, 2010). Quail in our study lost 11.6% of body weight during the 48-h simulated translocation, results consistent with weight loss in translocated starlings (5–15%) and wild valley quail (14.3%) in an actual translocation of similar duration (Reyna *et al.,* 2020), indicating that translocation weight loss may be a result of a chronic stress response to handling and captivity. Similar to our study, Reyna *et al.* (2020) reported that heavier valley quail lost less weight than lighter valley quail during a translocation. Heavier birds may be healthier overall, and future studies may benefit from considering a minimum weight for translocation.

By collecting faecal samples 4–5 times daily during the simulated translocation, we were able to capture diurnal variation in FCM concentrations. Although not previously observed in new world quail (Mohlman *et al.,* 2020), diurnal variation in CORT metabolites has been recorded in a variety of species (Florant and Weitzman, 1980; de Jong *et al.,* 2001; Touma *et al.,* 2004; Bosson *et al.,* 2009; Sheriff *et al.,* 2009), generally corresponding to the circadian rhythm of the animal. The diurnal variation in valley quail FCM concentrations followed a pattern similar to the variation in plasma CORT observed in broiler chickens (de Jong *et al.,* 2001), with lower concentrations at night and elevated concentrations during the day. This trend was expected since valley quail are active in the day and inactive during the night (Leopold, 1977).

This study verifies that translocations are stressful events for quail that result in significant weight loss, results that suggest stress influences translocation success. Chronic stress is associated with increased mortality in starlings (Cyr and Romero, 2007; Dickens *et al.,* 2010), and animal weight at release is a predictor of survival in cirl buntings (Fountain *et al.,* 2017). Further, increased CORT levels can inhibit reproduction, a primary indicator of translocation success (Griffith *et al.,* 1989; Dickens *et al.,* 2010). Future studies could benefit by focusing efforts on stress mitigation to increase long-term translocation success.

The use of FCM as a non-invasive measurement in new world quail could be extended beyond translocations to benefit quail conservation. There is considerable environmental and economic interest in conserving and restoring populations of quail and other gamebird species. Increased plasma CORT can be indicative of environmental disturbances and habitat-related metabolic challenges (Homyack, 2010; Shipley *et al.,* 2022). For example, FCMs have been used as an indicator of declining habitat quality in greater sage-grouse (*Centrocercus urophasianus*; Rabon *et al.,* 2021). FCMs have also been used to detect elevated CORT levels associated with extreme weather events, noise related to fossil fuel extraction (Cinto Mejia *et al.,* 2019) and other anthropogenic disturbances (Thiel *et al.,* 2008, 2011; Blickley *et al.,* 2012; Arlettaz *et al.,* 2015; Formenti *et al.,* 2015). Through the use of innovative field collection methods (Shipley *et al.,* 2019), FCMs may be used to assess chronic stress levels in wild quail populations, evaluate responses to multiple stressors (e.g. extreme drought) and map stress levels across a landscape to identify focus areas for conservation (Rabon *et al.,* 2021). Ultimately, the use of FCMs could alert quail biologists to the presence of environmental stressors before population demographics are impacted (Ellis *et al.,* 2012). Clearly, FCMs have enormous potential as a non-invasive indicator of stress levels in quail and as a tool for improving quail research, restoration, management and conservation.

## Data Availability

The data underlying this article will be shared on reasonable requests to the corresponding author.
